# Assessment of Deep Water-Saving Practice Effects on Crop Coefficients and Water Consumption Processes in Cultivated Land–Wasteland–Lake Systems of the Hetao Irrigation District

**DOI:** 10.3390/plants14182933

**Published:** 2025-09-21

**Authors:** Jiamin Li, Guoshuai Wang, Delong Tian, Hexiang Zheng, Haibin Shi, Zekun Li, Jie Ren, Ruiping Li

**Affiliations:** 1 College of Water Conservancy and Civil Engineering, Inner Mongolia Agricultural University, Hohhot 010018, China; ljm656@126.com (J.L.); shi_haibin@sohu.com (H.S.); 2 Institute of Water Resources for Pastoral Area Ministry of Water Resources, Hohhot 010020, China; imau_wgs@163.com (G.W.); mkstdl@126.com (D.T.); mkszhx@163.com (H.Z.); mkslzk@163.com (Z.L.); renj@iwhr.com (J.R.); 3 Yinshanbeilu Grassland Eco-Hydrology National Observation and Research Station, China Institute of Water Resources and Hydropower Research, Beijing 100038, China; 4 State Key Laboratory of Water Engineering Ecology and Environment in Arid Area, Inner Mongolia Agricultural University, Hohhot 010018, China

**Keywords:** Hetao Irrigation District, three representative plant types, cultivated land-wasteland-lake systems, SIMDualKc model, deep water-saving practice, crop coefficients, water consumption processes

## Abstract

Water scarcity, soil salinization, and desertification threaten sustainable agricultural ecosystems of Hetao irrigation district, Yellow River Basin (YRB). Precise quantification of soil water dynamics and plant water consumption processes is essential for the agricultural sustainability of the irrigation district. Therefore, this study mainly focused on the crop coefficients and water consumption processes of three representative plant types in the Hetao irrigation district, each corresponding to a specific land system: *Helianthus annuus* (cultivated land), *Tamarix chinensis* (wasteland), and *Phragmites australis* (lake). The SIMDualKc model was calibrated and validated based on situ observation data (soil water content and yield) during 2018 (conventional conditions), 2023 and 2024 (deep water-saving conditions). Results show strong agreement between simulated and observed soil moisture and crop yields. The results indicate that the process curves of *K_cb_* (basal crop coefficient) and *K_cb_*_adj_ (adjusted crop coefficient) nearly overlapped for the three plant types in 2018 and 2023. However, under the deep water-saving project implemented in 2024, the *K_cb_*_adj_ process curves for all three plant types exhibited a significant reduction (approximately 15%). Soil evaporation fractions (*E*/*ET_c_*_adj_) were stable at 19–30% during the 2018, 2023, and 2024. The contribution of capillary rise to *ET* reached 38.61–43.18% in cultivated land (*Helianthus annuus*), 41.52–48.93% in wasteland (*Tamarix chinensis*), and 38.08–46.57% in lake boundary areas (*Phragmites australis*), which underscores the significant role of groundwater recharge in sustaining plant water consumption. Actual-to-potential transpiration ratios (*T_a_*/*T_p_*) during 2023–2024 decreased by 3–11% for *Helianthus annuus*, 5–12% for *Tamarix chinensis*, and 23% for *Phragmites australis* compared to *T_a_*/*T_p_* values in 2018. Capillary rise decreased approximately 10% during the whole system. Deep water-saving practices increased the groundwater depth and restricted groundwater recharge to plants via capillary rise, thereby impairing plant transpiration and growth. These findings provide scientific support for sustainable agriculture and ecological security in the Yellow River Basin.

## 1. Introduction

The Hetao Irrigation District in Inner Mongolia, one of China’s three largest gravity-fed irrigation districts, relies entirely on irrigation for agriculture, making it a typical “no irrigation, no agriculture” region [1]. Following recent water-saving renovations, annual water diversion decreased from 5.2 billion m^3^ to 4.0 billion m^3^ [2], alleviating water stress but accelerating groundwater decline. The district covers 1.12 × 10^4^ km^2^, with cultivated land (5.74 × 10^3^ km^2^), saline–alkali land (2.09 × 10^3^ km^2^), and lakes (130 km^2^) as its three dominant land types [3]. Numerous small lakes are scattered across the region [4,5], with wasteland interspersed among cultivated fields, sand dunes, and lake margins [6,7]. Strong hydraulic connectivity exists between cultivated land, wasteland, and lakes [5]. In this arid climate, lake margins and wasteland exhibit high water consumption during the crop growing season, replenished mainly by rainfall and groundwater [8,9,10,11]. Dominant plants include *Helianthus annuus* and maize in cultivated fields, *Tamarix chinensis* in wastelands, and *Phragmites australis* in lake margins. Their water consumption acts as a critical link between the synergistic interaction among atmospheric water, soil water, and groundwater, thereby forming a complex agro-hydrological system that is driven by evapotranspiration, soil groundwater recharge, and surface water fluxes [12,13,14,15].

The SIMDualKc model [16,17,18], developed from the FAO-56 dual crop coefficient method, estimates daily crop water consumption based on water balance principles, while effectively partitioning transpiration and soil evaporation. Zhao et al. [19] applied SIMDualKc to simulate crop coefficients and soil evaporation for maize–wheat intercropping in North China. Yan et al. [20] reported evaporation fractions of 24.1–28.7% for drip-irrigated summer maize in semi-arid Northwest China using the model. Ren et al. [21] demonstrated that in shallow groundwater areas, approximately 40% of percolated irrigation water is reused by crops through capillary rise, with capillary contributions to evapotranspiration ranging from 23–53% in cultivated land and 75–82% in wasteland. Excessive irrigation and shallow groundwater caused about 40% of water in cultivated land and 50% in wasteland to be lost through evaporation during the main crop season (May–September), thereby exacerbating root-zone salt accumulation. Crop and natural vegetation primarily experience salt stress under conditions of sustained high root-zone moisture, with the stress mainly originating from accumulated salts. Wang et al. [22] investigated water transfer and transformation within the cultivated land–wasteland–lake system using a two-end-member mixing model based on hydrogen and oxygen isotopes and soil water dynamics. Their results revealed that irrigation water infiltrating to groundwater in cultivated areas through lateral subsurface flow is largely transferred to groundwater in wasteland zones, with this process primarily driven by canal-sourced lateral subsurface flow. Li et al. [23], based on SEBAL model inversion in the Yongji subdistrict of the Hetao Irrigation District, found that daily evapotranspiration followed the order of water body > cultivated land > wasteland. Yang et al. [24] further reported that groundwater depth in the Shenwu subdistrict increased significantly after water-saving renovations, with mean groundwater depth rising by 83.9% compared to pre-renovation levels. Existing hydrological studies have mainly concentrated on crop water consumption in cultivated land, while ecohydrological processes in natural vegetation remain largely overlooked. Systematic understanding of hydraulic linkages across cultivated land (*Helianthus annuus*), wasteland (*Tamarix chinensis*), and lake margins (*Phragmites australis*) is still limited. Yet, vegetation growth and water consumption are integral to ecohydrological processes. The deep water-saving project is a comprehensive water resource management strategy for arid and semi-arid regions. Its core lies in constructing efficient water-saving irrigation projects and implementing refined management measures. On the basis of significantly reducing the total agricultural water consumption and optimizing the water use structure, it promotes the regional groundwater depth (GWD) to rise and stabilize within the “ecological safety range”, ultimately achieving a win–win situation for agricultural development and ecological protection. Moreover, recent implementation of deep water-saving measures has profoundly altered hydrological cycles not only in irrigated farmland but also in wasteland and lake systems—an urgent scientific issue.

This study hypothesizes that the SIMDualKc model, after localized parameter calibration, can reliably simulate crop coefficient dynamics and water consumption processes of *Helianthus annuus*, *Tamarix chinensis*, and *Phragmites australis* in the Hetao Irrigation District. Furthermore, deep water-saving projects and groundwater decline not only reduce water consumption in cultivated land but also significantly alter water use processes of wasteland and lake vegetation by modifying hydraulic linkages, potentially leading to shifts in system water–salt balance and ecological patterns.

Therefore, the main objective of this study is to simulate crop coefficients and water consumption of three representative plants within the cultivated land–wasteland–lake system of the Hetao Irrigation District, and to evaluate the effects of deep water-saving measures and groundwater decline on their dynamics. Findings provide essential theoretical support for efficient agricultural water use and ecohydrological security in the Yellow River Basin.

## 2. Materials and Methods

### 2.1. Overview of the Study Area

**Figure 1:**
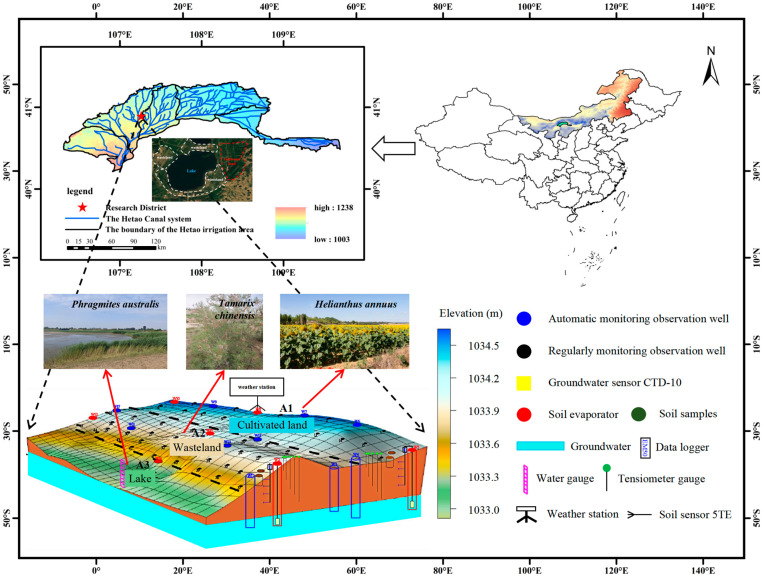
Layout of the experimental setup.

**Figure 2:**
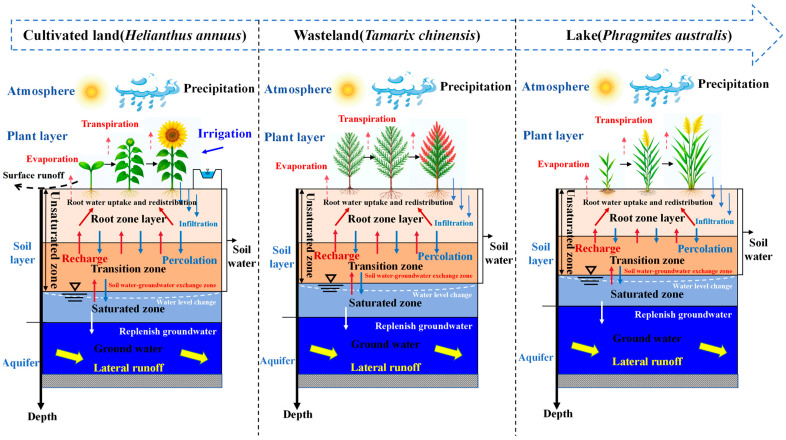
Description of plant water consumption process in cultivated land–wasteland–lake systems.

**Figure 3:**
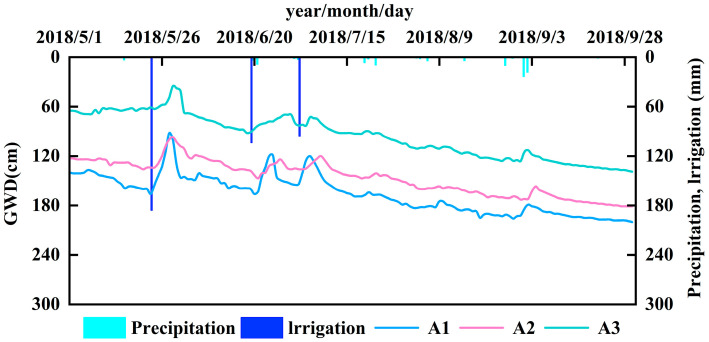

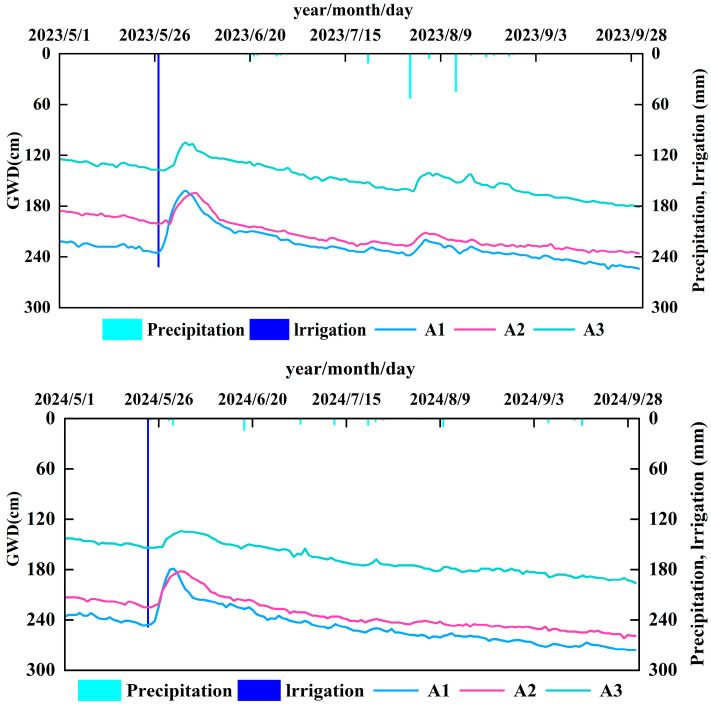
Groundwater depth variation.

**Figure 4:**
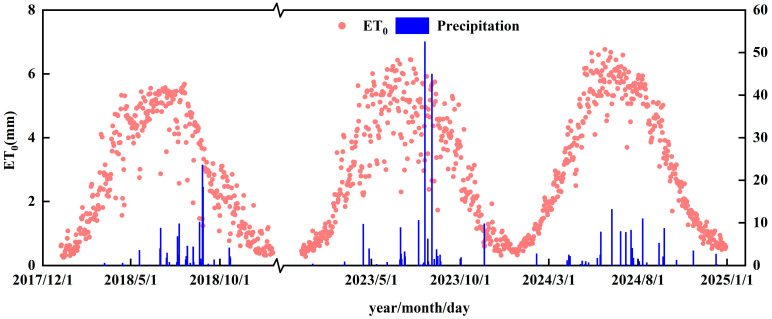
Rainfall and *ET*_0_ variations in 2018, 2023, and 2024.

The study area is located at Zhangliansheng Lake (40°54′36.24″ N, 107°15′59.07″ E; elevation 1035 m) in the Jiefangzha Irrigation District of the Hetao Irrigation District, Inner Mongolia. The experimental site comprises approximately 8 ha of cultivated land and wasteland, adjacent to a 51.2 ha lake (Figure 1) [25]. The region has a multi-year average temperature of 8.5 °C and a frost-free period of 130–150 days. Figure 2 illustrates the water consumption processes across the three land types. RTK surveys indicated a maximum elevation difference of 15 cm across farmland, with cultivated land averaging 45 cm higher than wasteland. During the growing season, the groundwater depth in the experimental area varied as follows: cultivated land (1.30–2.60 m), wasteland (1.30–2.40 m), and lake boundary (1.00–1.80 m) (Figure 3), with an average of 1.80 m. Precipitation was concentrated in June–August, with growing season totals of 113.4 mm (2018), 149.1 mm (2023), and 86.8 mm (2024) (Figure 4). The study area comprised cultivated land, wasteland, and the lake area, where *Helianthus annuus*—sown in early June and harvested in early October with a 128-day growing season—was the primary crop, while salt-tolerant vegetation, primarily *Tamarix chinensis* and *Phragmites australis*, occupied the wasteland and lake boundary zones. The soil texture within the 0–300 cm profile of the study area is predominantly sand and sandy loam (Table 1). According to the WRB 2022 classification, soils at the typical sampling points are mainly Arenosols, with the surface layer locally identified as Loamic Arenosols and the deeper layers characterized as Haplic Arenosols (Arenic) [26].

### 2.2. Experimental Setup and Data Collection

#### 2.2.1. Monitoring of Soil Water and Salt

The study area comprised a total of 63 general soil observation points spaced at 50 m intervals. Soil sampling at these points was conducted to a depth of 100 cm, divided into five layers at 20 cm intervals. Soil samples were collected at 10-day intervals, with intensified sampling before and after irrigation events. Soil water content was determined by the gravimetric method (oven-drying), and the electrical conductivity of soil extracts (prepared at a soil/water ratio of 1:5) was measured using a conductivity meter (DDS-307A, Shanghai Youke Instrument Co., Ltd., Shanghai, China). Additionally, seven intensive soil observation points were established, where soil sampling extended to a depth of 200 cm, also divided into layers at 20 cm intervals. At these intensive points, automated soil sensors (5TE, METER Group, Inc., Pullman, WA, USA) were installed to measure soil moisture and salinity, with data recorded using an EM50 data logger [27,28,29].

#### 2.2.2. Groundwater Monitoring

A total of 17 groundwater monitoring wells were installed within the study area, comprising 7 intensive monitoring wells and 10 general monitoring wells. Automated groundwater sensors (CTD-10, METER Group, Inc., USA) were installed in the intensive wells, with groundwater level and salinity recorded hourly by EM50 data loggers. For the general wells, groundwater table depth was measured every 7 days, and water samples were collected every 10 days for salinity analysis. Three key intensive monitoring sites were selected: A1 (cultivated land—*Helianthus annuus*), A2 (wasteland—*Tamarix chinensis*), and A3 (lake boundary—*Phragmites australis*) (Figure 1). The groundwater level variations at these sites are presented in Figure 3, exhibiting similar fluctuation patterns.

#### 2.2.3. Meteorological Data

Daily meteorological data, including air temperature, relative humidity, sunshine hours, wind speed, and precipitation, were collected from the weather station within the study area. Utilizing these daily meteorological data, the reference evapotranspiration (*ET*_0_) was calculated for each day using the Penman–Monteith equation [30]. The *ET*_0_ and precipitation data for 2018, 2023, and 2024 are presented in Figure 4. The total annual precipitation for 2018, 2023, and 2024 was 124 mm, 176.1 mm, and 105.1 mm, respectively. The mean daily *ET*_0_ over the entire year for these years was 2.8 mm/d, 2.95 mm/d, and 3.22 mm/d, respectively. During the crop growing season, the mean daily *ET*_0_ was 4.25 mm/d, 4.62 mm/d, and 5.2 mm/d, respectively.

#### 2.2.4. Plant Monitoring

*Helianthus annuus* was irrigated, whereas *Tamarix chinensis* and *Phragmites australis* received no irrigation. *Helianthus annuus* was irrigated three times in 2018. Following the implementation of deep water-saving measures, the irrigation frequency was reduced to once annually in 2023 and 2024 (Table 2). The total dissolved solids (TDS) fluctuated between 0.57 and 0.61 g L^−1^, and reference [31] provides details on the TDS calculation method. Plant heights for *Helianthus annuus* (the crop) and the natural vegetation species during their key growth stages are presented in Table 3.

### 2.3. Research Methods

#### 2.3.1. Introduction to the Dual Crop Coefficient Model

The FAO-56 dual crop coefficient model partitions the crop coefficient (*K_c_*), used to calculate crop evapotranspiration (*ET_c_*) under standard conditions, into a basal crop coefficient (*K_cb_*) representing plant transpiration and a soil evaporation coefficient (*Ke*) representing soil evaporation: *ET_c_* = (*K_cb_* + *K_e_*) *ET*_0_. Furthermore, it incorporates daily water balance computations for both the root zone and the soil evaporation layer to determine water stress and soil evaporation reduction [32,33,34].

The model employs the FAO Penman–Monteith equation to calculate reference evapotranspiration (*ET*_0_).

(1) $${ET}_{0}=\frac{0.408\Delta \left(R_{n}-G\right)+\gamma \frac{900u_{2}}{T+273}\left(e_{s}-e_{a}\right)}{\Delta +\gamma \left(1+0.34u_{2}\right)}$$

In the equation, *ET*_0_ is the reference crop evapotranspiration (mm·d^−1^); *R_n_* is the net radiation (MJ·m^2^·d^−1^); *G* is the soil heat flux density (MJ·m^2^·d^−1^); *γ* is the psychrometric constant (kPa·°C^−1^); *u*_2_ is the wind speed at 2 m height (m·s^−1^); *T* is the mean daily air temperature at 2 m height (°C); *e_s_* is the saturation vapor pressure (kPa); *e_a_* is the actual vapor pressure (kPa); Δ is the slope of the vapor pressure curve (kPa·°C^−1^).

The basal crop coefficient (*K_cb_*) is the ratio of crop evapotranspiration (*ET_c_*) to reference evapotranspiration (*ET*_0_) (*ET_c_*/*ET*_0_) under conditions where crop transpiration occurs at a potential rate without soil evaporation (where the soil surface is dry, but sufficient moisture is available to sustain crop transpiration). Consequently, the potential vegetation transpiration rate (*T_p_*) can be expressed by the following equation:

(2) $$T_{P}=K_{cb}{ET}_{0}$$

In the equation, *ET*_0_ is calculated using the Penman–Monteith equation recommended by the Food and Agriculture Organization of the United Nations (FAO). Under semi-humid climatic conditions and moderate wind speed, recommended *K_cb_* values for different crops are listed in Table 17 of FAO56. When *RH_min_* is not 45% or wind speed is not 2 m s^−1^, *K_cb_*_mid_ and *K_cb_*_end_ greater than 0.45 require correction using the following equation:

(3) $$K_{cb}=K_{cb\left(tab\right)}+\left[0.04\left(u_{2}-2\right)-0.004\left({RH}_{min}-45\right)\right]{\left(\frac{h}{3}\right)}^{0.3}$$

In the equation, *K_cb(tab)_* refers to the *K_cb_*_mid_ and *K_cb_*_end_ values obtained from Table 17 of FAO56 (if ≥0.45); *u*_2_ is the mean wind speed at 2 m height during the mid- and late-season crop growth stages (m s^−1^), with 1 m s^−1^ ≤ *u*_2_ ≤ 6 m s^−1^; *RH _min_* is the mean minimum relative humidity during the mid- and late-season growth stages (%), with 20% ≤ *u*_2_ ≤ 80%; h is the mean plant height during the mid- and late-season growth stages (m).

The soil evaporation coefficient (*K_e_*) relates to the wetness of the topsoil layer. *K_e_* increases with higher topsoil moisture content, whereas it decreases to near zero when the topsoil becomes dry. The calculation formula for *K_e_* is as follows:

(4) $$K_{e}=K_{r}\left(K_{c\;max}-K_{cb}\right)\leq f_{ew}K_{c\;max}$$

In the equation, *Kᵣ* is the evaporation reduction coefficient dependent on the accumulated evaporation depth from the topsoil layer (dimensionless); *K_c max_* is the maximum value of *K_c_* following rainfall or irrigation; *f_ew_* is the fraction of soil surface exposed to evaporation (%).

The evaporation reduction coefficient (*K_r_*) occurs in two distinct stages: Stage 1 is the energy-limited stage, and Stage 2 is the soil moisture-limited stage. The calculation formulas for the two stages of *K_r_* are as follows:

(5) $$K_{r}=1\left(D_{e,\;i-1}\leq REW\right)$$

(6) $$K_{r}=\frac{TEW-D_{e,i-1}}{TEW-REW}\left(REW<D_{e,i-1}\right)$$

In the equation, TEW is the maximum cumulative evaporation depth from the topsoil when fully wet (mm); *D*_*e*,*i*−1_ is the cumulative evaporation depth from the soil evaporation layer at the end of day i − 1 (mm); REW is the cumulative evaporation depth for Stage 1 (mm).

The water stress coefficient (*K_s_*) quantifies the effect of water stress on crop transpiration, with 0 < *K_s_* ≤ 1. Lower *K_s_* values indicate greater water stress effects. *K_s_* = 1 denotes no water stress, while *K_s_* approaching 0 signifies severe water stress. The *K_s_* calculation formula is

(7) $$K_{s}=\frac{TAW-D_{r,i}}{TAW-RAW}$$

In the equation, TAW and RAW are the total available water and readily available water in the root zone (mm), respectively; *D_r,i_* is the root zone water depletion on day i (mm).

The above equations provide a systematic method for calculating *K_cb_* under potential conditions. When vegetation growth is subjected to stress, it is necessary to further correct *K_cb_* by multiplying it with the stress coefficient *K_s_*.

#### 2.3.2. Model Construction

##### Meteorological Database

Meteorological data were automatically collected hourly from January to December during 2018 and 2023–2024. Primary meteorological parameters included the mean relative humidity (*RH_mean_*, %), minimum relative humidity (*RH_min_*, %), maximum air temperature (*T_max_*, °C), minimum air temperature (*T_min_*, °C), sunshine duration (*n*, h), wind speed at 2 m height (*u*_2_, m s^−1^), and precipitation (mm). Daily reference evapotranspiration (*ET*_0_) was calculated using the FAO Penman–Monteith equation with these parameters.

##### Field Management Database

Since early May, systematic monitoring was conducted on *Helianthus annuus*, *Tamarix chinensis*, and *Phragmites australis* within the experimental area. The observations covered the entire growth period, including changes in plant height and root depth, with precise documentation of the dates corresponding to each key developmental stage. Following local farming practices, the irrigation dates and amounts for *Helianthus annuus* were recorded. Field management practices, including weeding, fertilization, soil loosening, pesticide application, and pest/disease control, were implemented and documented in real-time.

##### Initial Conditions

(1) Soil data: Field capacity (*θ_fc_*, %), permanent wilting point (*θ_wp_*, %), soil layer depth specifications, total evaporable water (TEW, mm), readily evaporable water (REW, mm), and total available water (TAW, mm m^−1^).
(2) Meteorological data: Minimum relative humidity (*RH_min_*, %), maximum temperature (*T_max_*, °C), minimum temperature (*T_min_*, °C), wind speed at 2 m height (m s^−1^), reference evapotranspiration (*ET*_0_, mm), and precipitation (*P*, mm).
(3) Crop data: Sowing dates, growth stage divisions/durations, maximum root depth (m), plant height (m), basal crop coefficients, and leaf area index.
(4) Irrigation data: Irrigation dates, amounts, and maximum application depth.
(5) Optional inputs: Mulching status, surface runoff, deep percolation, and soil salinity.
(6) The SIMDualKc model also accounts for plant water consumption processes within cultivated land–wasteland–lake systems, as illustrated in Figure 2.

### 2.4. Calibration and Validation

Readily Evaporable Water (REW) and Total Evaporable Water (TEW) are two key parameters that characterize soil water movement and crop response processes. Conducting a sensitivity analysis on these parameters helps identify the sources of uncertainty in model outputs. The analysis of REW and TEW reveals that REW primarily and sensitively controls the onset of water stress, as well as the partitioning ratio of early-stage evaporation and transpiration in total evapotranspiration (*ET*); its value directly affects the sustainability of water available to crops at the initial stage of drought. In contrast, TEW sensitively regulates soil water buffering capacity throughout the entire growing season, significantly influencing the frequency and intensity of water stress, along with the potential risk of deep percolation. Together, these two parameters determine the water dynamics and water use efficiency of the soil–plant system, and they are sensitive variables requiring focused attention during model calibration and validation.

To evaluate the accuracy of model simulations, this study employed the coefficient of determination (*R^2^*), root mean square error (*RMSE*), and Nash–Sutcliffe efficiency (*NSE*) as evaluation metrics:

(8) $$R^{2}=\left[\frac{\sum_{i=1}^{N}\left(O_{i}-\bar{O}\right)\left(P_{i}-\bar{P}\right)}{{\left[\sum_{i=1}^{N}{\left(O_{i}-\bar{O}\right)}^{2}\right]}^{0.5}{\left[\sum_{i=1}^{N}{\left(P_{i}-\bar{P}\right)}^{2}\right]}^{0.5}}\right]$$

(9) $$RMSE={\left[\frac{\sum_{i=1}^{N}{\left(P_{i}-O_{i}\right)}^{2}}{n}\right]}^{0.5}$$

(10) $$NSE=1-\frac{\sum_{i=1}^{N}{\left(P_{i}-O_{i}\right)}^{2}}{\sum_{i=1}^{N}{\left(O_{i}-\bar{O_{i}}\right)}^{2}}$$

where *N* is the number of measured values, *O*_i_ is the i-th measured value, *P_i_* is the corresponding i-th simulated value, *Ō* is the mean of observed values, and $\bar{P}$ is the mean of simulated values. *RMSE* approaching 0 indicates higher simulation accuracy. The *NSE* ranges from −∞ to 1, where *NSE* = 1 signifies perfect agreement between simulated and measured values. Negative *NSE* values indicate that model predictions are less accurate than using the observed mean. *R^2^* approaching 1 demonstrates the model’s strong capability to capture variability in measured data. Generally, the ratio of *RMSE* to the average observed value should be ≤20%, with *R^2^* ≥ 0.5 to meet calibration requirements.

## 3. Results and Analysis

### 3.1. Model Calibration and Validation

#### 3.1.1. Model Calibration

**Figure 5:**
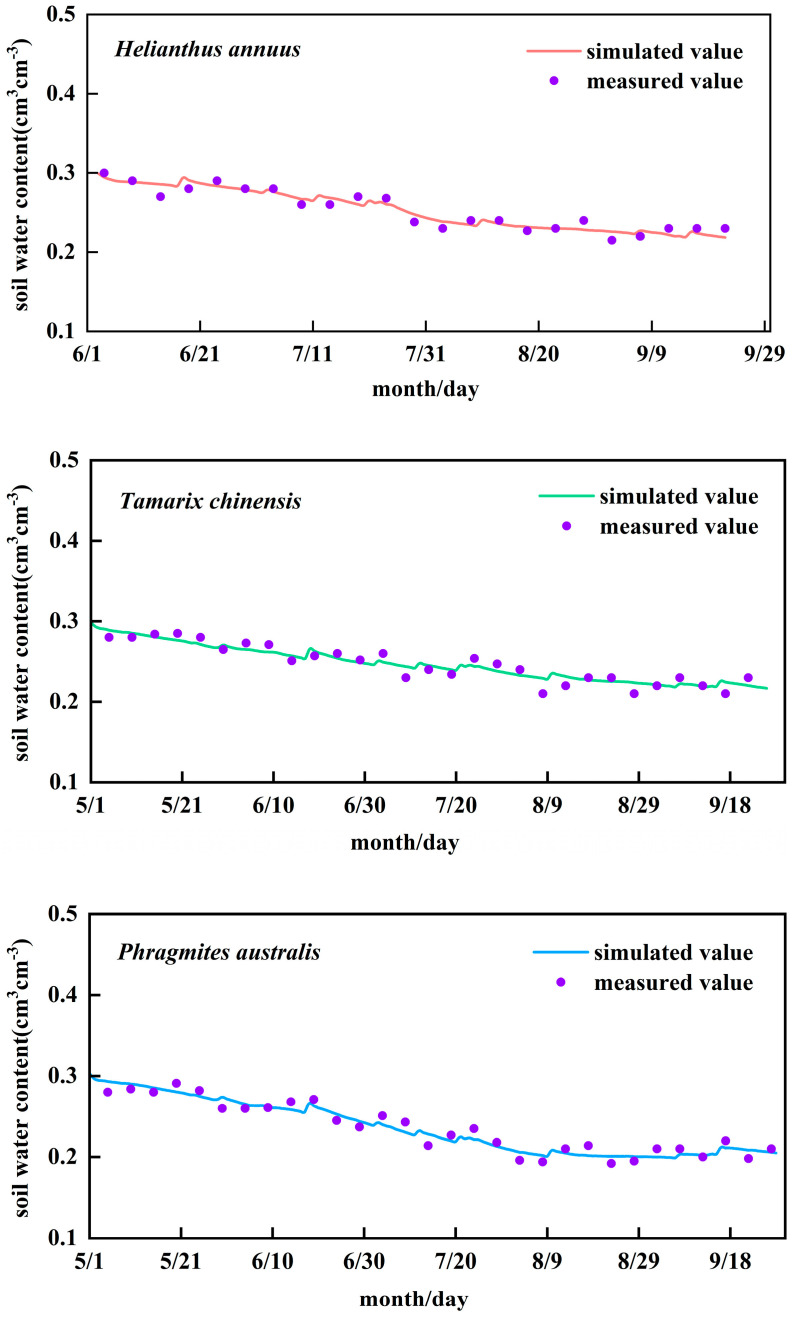
Simulated versus measured soil water contents during model calibration.

During the model calibration process (2024), the comparison between the simulated and measured values of soil water content is shown in Figure 5. The results demonstrate good agreement between simulated and measured soil moisture, with simulations accurately capturing the temporal dynamics of soil water. For cultivated *Helianthus annuus*, *Tamarix chinensis*, and *Phragmites australis*, *RMSE* values were approximately 0.01 cm^3^ cm^−3^, while *R^2^* and *NSE* exceeded 0.80 (Table 4). The ratio of *RMSE* to the average observed value for three typical plants is within 5%, thus meeting the calibration requirements. The relative error between simulated and measured *Helianthus annuus* yield was 10.3%, indicating sufficient model accuracy for this study. Soil evaporation parameters are presented in Table 5. The calibrated readily evaporable water (REW) for wildland was significantly lower than FAO-56 recommendations, likely due to salt efflorescence and crust formation inhibiting soil evaporation after prolonged exposure.

#### 3.1.2. Model Validation

**Figure 6:**
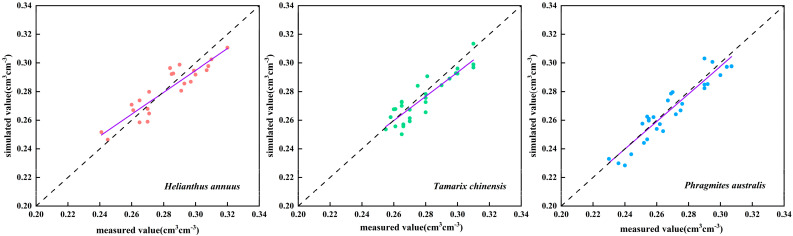
Comparison of simulated and measured soil water contents during model validation for crops.

Model validation was performed using 2024 field observation data with identical parameters and empirical coefficients from the calibration phase. The results demonstrate favorable performance metrics for soil moisture: *RMSE* (0.01 cm^3^ cm^−3^), *R^2^* (0.81–0.87), and *NSE* (0.78–0.85) (Table 4), confirming that the calibrated model effectively replicates field-observed soil water dynamics. During validation, *Helianthus annuus* exhibited lower *R^2^* and *NSE* values compared to calibration, primarily attributed to heightened data variability from frequent heavy rainfall events in 2024 (Figure 6). The relative error between simulated and measured *Helianthus annuus* yield was 4.8% during validation (Table 6). These findings collectively indicate the model’s robust capability to simulate soil water dynamics and flux processes, establishing its suitability for agricultural hydrological analysis and irrigation water use efficiency assessments.

### 3.2. Crop Parameters and Water Consumption Characteristics of Cultivated Helianthus annuus

**Figure 7:**
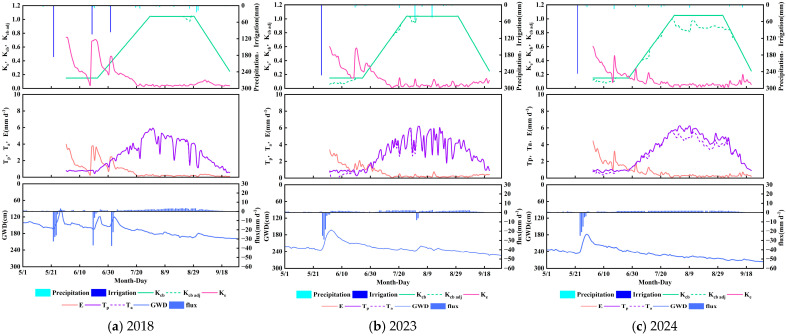
Crop coefficients (*K_cb_*, *K_cb_*_adj_, and *K_e_*), potential (*T_p_)* and actual transpiration (*T_a_*), soil evaporation (*E*) and vertical bottom flux at 90 cm soil profile versus irrigation, precipitation, and groundwater depth (GWD) changes during the simulation period for *Helianthus annuus* in 2018 (**a**), 2023 (**b**), and 2024 (**c**).

As illustrated in Figure 7, *Helianthus annuus*—a typical annual crop in saline–alkali soils—exhibits water consumption patterns co-regulated by dynamic basal crop coefficient (*K_cb_*) variations and irrigation management. The *K_cb_* values during the initial (*K_cb_*_ini_), mid-season (*K_cb_*_mid_), and late-season (*K_cb_*_end_) stages were 0.15, 1.04, and 0.25, respectively, aligning closely with FAO-56 recommendations. This “low–high–low” trajectory reflects stage-specific transpiration demands: limited canopy development in initial stages, peak water demand during vigorous mid-season growth, and reduced transpiration during late-season senescence. The adjusted crop coefficient (*K_cb_*_adj_), modified for water–salt stress, closely tracked *K_cb_* in 2018 and 2023, with actual-to-potential transpiration ratios (*T_a_*/*T_p_*) reaching 100% and 97%, indicating optimal conditions. In contrast, 2024 showed significant *K_cb_*_adj_ reduction during the late season (*T_a_*/*T_p_* = 89%), signaling enhanced stress from salt accumulation or water deficit.

The soil evaporation coefficient (*K_e_*) peaked during initial growth due to low canopy cover, reaching 0.74 (2018), 0.60 (2023), and 0.61 (2024), particularly post-rainfall/irrigation when solar radiation and aerodynamic forces drove rapid topsoil evaporation. *K_e_* declined below 0.05 as canopy cover increased, with minor late-season rebounds (0.09–0.12) limited by deep soil moisture depletion. Evaporation’s contribution to total evapotranspiration (*E*/*ET_c_*_adj_) ranged between 19 and 23%, consistent with the existing literature [35,36].

Evapotranspiration exhibited negligible interannual differences, with values of 403 mm (2018), 395 mm (2023), and 404 mm (2024). Deep percolation measured 198 mm, 94 mm, and 70 mm across the three years, accounting for 51.30%, 37.15%, and 28.34% of irrigation input, respectively. Capillary rise reached 174 mm, 160 mm, and 156 mm, contributing 38.61–43.18% to the total *ET* In 2018, cumulative capillary rise was lower than deep percolation, yielding a net downward flux of 24 mm. By contrast, 2023 and 2024 showed greater capillary rise than deep percolation, generating net upward fluxes of 66 mm and 113 mm, respectively, emphasizing groundwater’s contribution to root-zone replenishment. Under intensified water-saving practices (2023–2024), substantially reduced irrigation compared to conventional 2018 levels reversed net flux direction from downward to upward. Although 2023 storms elevated water tables and enhanced capillary rise, increased canopy cover effectively suppressed soil evaporation, achieving concurrent high *ET* and low evaporation. During 2024 drought, capillary rise remained stable at 156 mm, demonstrating groundwater’s critical compensatory mechanism against drought stress.

### 3.3. Crop Coefficient and Water Consumption Characteristics of Wildland Tamarix chinensis

**Figure 8:**
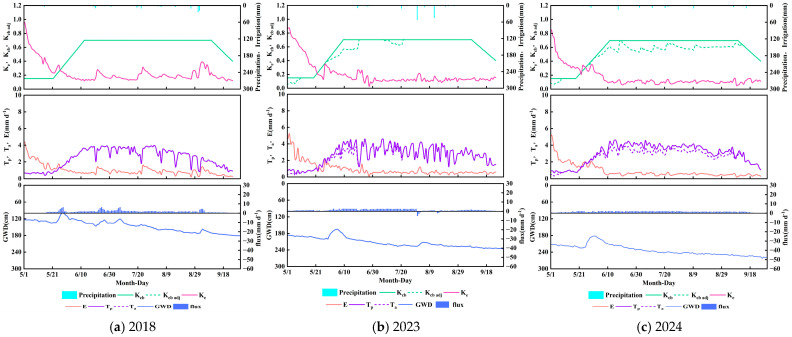
Crop coefficients (*K_cb_*, *K_cb_*_adj_ and *K_e_*), potential (*T_p_*) and actual transpiration (*T_a_*), soil evaporation (*E*), and vertical bottom flux at 90 cm soil profile versus irrigation, precipitation, and groundwater depth (GWD) changes during the simulation period for *Tamarix chinensis* in 2018 (**a**), 2023 (**b**), and 2024 (**c**).

As shown in Figure 8, *Tamarix chinensis*—a perennial salt-tolerant shrub—exhibited basal crop coefficients (*K_cb_*) of 0.15 (initial), 0.7 (mid-season), and 0.4 (late season). Transpiration demand peaked during mid-season but remained consistently lower than annual crops (e.g., *Helianthus annuus*). In 2018 and 2023, the adjusted crop coefficient (*K_cb_*_adj_) closely aligned with *K_cb_*, with actual-to-potential transpiration ratios (*T_a_*/*T_p_*) reaching 100% and 95%, indicating minimal water–salt stress. The 2024 *K_cb_*_adj_ profile declined significantly (*T_a_*/*T_p_* = 88%), likely due to groundwater table decline and surface salt accumulation.

The soil evaporation coefficient (*K_e_*) of wildland *Tamarix chinensis* exhibited a trend similar to croplands, showing elevated values when the groundwater table was high or after rainfall events. This primarily occurs because solar radiation and aerodynamic forcing drive rapid evaporation of moisture from the topsoil layer. During initial growth, *K_e_* temporarily increased to approximately 0.9 due to groundwater table elevation from adjacent field irrigation. *K_e_* gradually decreased to approximately 0.13 with canopy development during peak growth. Despite reduced canopy cover in late season, *K_e_* showed no significant rebound due to rapid topsoil desiccation from irrigation absence and groundwater table decline.

Evapotranspiration under adjusted conditions (*ET_c_*_adj_) for wildland *Tamarix chinensis* measured 515 mm (2018), 516 mm (2023), and 525 mm (2024), with the evaporation-to-evapotranspiration ratio (*E*/*ET_c_*_adj_) maintained between 26 and 30%. Although total evapotranspiration exceeded that of croplands, soil evaporation was significantly greater. Global studies on evapotranspiration partitioning under varying cover conditions indicate soil evaporation typically constitutes 20–40% of total *ET* [37,38], confirming wildland evaporation fell within normal ranges. Deep percolation occurred only during the 2023 storm event, accumulating merely 13 mm. Capillary rise reached 252 mm, 217 mm, and 218 mm across the three years, contributing 41.52–48.93% to total *ET*. Cumulative capillary rise consistently exceeded deep percolation, yielding net upward fluxes of 252 mm, 204 mm, and 218 mm, demonstrating sustained groundwater replenishment to roots via capillarity. Under intensified water-saving practices (2023–2024), groundwater table decline reduced lateral moisture transfer, exacerbating water stress in wildland *Tamarix chinensis* and decreasing capillary rise by approximately 13.7%. Nevertheless, *Tamarix chinensis* maintained high transpiration efficiency through its salt-tolerance adaptation, highlighting its ecological resilience advantage.

### 3.4. Crop Coefficient and Water Consumption Characteristics of Phragmites australis at the Lake Boundary

**Figure 9:**
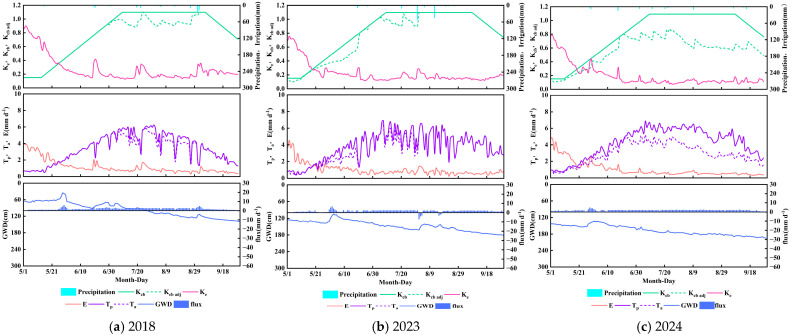
Crop coefficients (*K_cb_*, *K_cb_*_adj_, and *K_e_*), potential *(T_p_*) and actual transpiration (*T_a_*), soil evaporation (*E*), and vertical bottom flux at 90 cm soil profile versus irrigation, precipitation, and groundwater depth (GWD) changes during the simulation period for *Phragmites australis* in 2018 (**a**), 2023 (**b**), and 2024 (**c**).

As shown in Figure 9 *Phragmites australis*, the dominant vegetation in wetland–saltmarsh transition zones, exhibited basal crop coefficients (*K_cb_*) of 0.15 (initial), 1.1 (mid-season), and 0.7 (late season). Its mid-season transpiration demand was substantially higher than *Helianthus annuus* and *Tamarix chinensis*, reflecting high biomass production and wetland adaptation. During 2018 and 2023, the adjusted crop coefficient (*K_cb_*_adj_) closely aligned with *K_cb_*, with actual-to-potential transpiration ratios (*T_a_*/*T_p_*) averaging 93%. In 2024, *K_cb_*_adj_ significantly declined (*T_a_*/*T_p_* = 70%), indicating enhanced stress from groundwater table decline and surface salt accumulation.

The soil evaporation coefficient (*K_e_*) temporarily increased during initial growth due to surface wetness, peaking at 0.9 (2018), 0.76 (2023), and 0.79 (2024). As the canopy rapidly developed, effective ground shading reduced *K_e_* below 0.17. Notably, despite canopy senescence in late season, *K_e_* showed no expected rebound due to diminished capillary rise from persistent groundwater table decline.

Evapotranspiration under adjusted conditions (*ET_c_*_adj_) for lakeside *Phragmites australis* measured 642 mm (2018), 688 mm (2023), and 603 mm (2024), with the evaporation-to-evapotranspiration ratio (*E*/*ET_c_*_adj_) maintained between 23 and 26%. Deep percolation occurred only during the 2023 storm event, accumulating 20 mm. Capillary rise reached 299 mm, 262 mm, and 266 mm across the three years, contributing 38.08–46.57% to total *ET*. Cumulative capillary rise consistently exceeded deep percolation, yielding net upward fluxes of 299 mm, 242 mm, and 266 mm, demonstrating groundwater’s dominant role in water supply. Under intensified water-saving practices (2023–2024), groundwater table decline reduced lateral moisture transfer, exacerbating water stress in lakeside *Phragmites australis* and decreasing capillary rise by approximately 11.7%, with the actual-to-potential transpiration ratio (*T_a_*/*T_p_*) declining by about 23%. Nevertheless, capillary rise in this zone remained significantly higher than in croplands and wildlands. This is directly attributable to *Phragmites australis*’s deep root system, which enables efficient groundwater utilization.

### 3.5. Analysis of Differences in Crop Coefficients and Water Consumption Characteristics Among Plant Types

As illustrated in Figure 7, Figure 8 and Figure 9, significant differences exist in crop coefficients and water consumption patterns across plant types. *Helianthus annuus* exhibited the characteristic low–high–low basal crop coefficient (*K_cb_*) dynamic: 0.15 at the initial stage, peak of 1.04 at mid-season, and 0.25 at late season. *Tamarix chinensis* showed the lowest mid-season peak *K_cb_* at 0.7. *Phragmites australis* demonstrated the highest mid-season *K_cb_* peak of 1.1, reflecting distinct ecological adaptations to saline environments.

Peak soil evaporation coefficients (*K_e_*) differed substantially during 2018–2024: cultivated *Helianthus annuus* ranged from 0.60 to 0.74, wildland *Tamarix chinensis* stabilized near 0.90, and lakeside *Phragmites australis* varied from 0.76 to 0.90. Under conventional conditions (2018), all three systems reached maximum *K_e_* values. *Tamarix chinensis*’s elevated evaporation was driven by irrigation from adjacent croplands, while *Phragmites australis* maintained high evaporation due to proximity to lakes.

The evaporation-to-evapotranspiration ratio (*E*/*ET_c_*_adj_) displayed a clear gradient: highest in wildland *Tamarix chinensis* (26–30%), lowest in cultivated *Helianthus annuus* (19–23%), with intermediate values in *Phragmites australis* (23–26%). Total evapotranspiration ranked *Phragmites australis* highest (603–688 mm), *Tamarix chinensis* intermediate (515–525 mm), and *Helianthus annuus* lowest (395–404 mm). Capillary rise followed *Phragmites australis* (262–299 mm) > *Tamarix chinensis* (217–252 mm) > *Helianthus annuus* (156–174 mm), with contribution rates to *ET* at 41.5–48.9% (*Tamarix chinensis*), 38.1–46.6% (*Phragmites australis*), and 38.6–43.2% (*Helianthus annuus*). Net water flux direction revealed *Tamarix chinensis* and *Phragmites australis* persistently relied on groundwater recharge, while *Helianthus annuus* shifted from net downward to upward flux after water-saving implementation. Three divergent water-use strategies emerged: *Helianthus annuus* achieved water redistribution through irrigation management; *Tamarix chinensis* maintained transpiration efficiency via groundwater access and salt tolerance; *Phragmites australis* exhibited high consumption capacity but extreme sensitivity to groundwater decline.

### 3.6. Impacts of Intensified Water-Saving Practices and Groundwater Decline

During the 2018 and 2023 growing seasons (1 May to 30 September), the average groundwater levels at sites A1, A2, and A3 declined by 61 cm, 66 cm, and 51 cm, respectively. During the 2023 and 2024 growing seasons (1 May to 30 September), the average groundwater levels at sites A1, A2, and A3 declined by 21 cm, 22 cm, and 20 cm, respectively.

During 2018 and 2023, adjusted crop coefficients (*K_cb_*_adj_) for all plants closely aligned with *K_cb_* process lines, indicating minimal water–salt stress. Under 2024 intensified water-saving conditions, significant *K_cb_*_adj_ reduction occurred (decrease of approximately 15%), reflecting enhanced stress from groundwater table decline and surface salt accumulation.

Intensified water-saving practices and groundwater depletion fundamentally altered water consumption patterns. Irrigation reduction (approximately 35% in croplands) led to greater groundwater dependence: *Helianthus annuus*’s capillary rise decreased by approximately 10.2%, yet maintained 38.6–43.2% contribution to *ET*, with net flux shifting from downward to upward; *Tamarix chinensis* and *Phragmites australis* showed 13.7% and 11.7% reductions in capillary rise, respectively, but retained high contribution rates (41.5–48.9% and 38.1–46.6%) under persistent net upward flux dominance.

Groundwater decline exacerbated water stress, directly reducing transpiration efficiency: *Helianthus annuus*’s actual-to-potential transpiration ratio (*T_a_*/*T_p_*) decreased by 3–11%, *Tamarix chinensis* by 5–12%, and *Phragmites australis* dramatically by 23%. Evaporative fractions varied concurrently, with *Tamarix chinensis* maintaining the highest *E*/*ET_c_*_adj_ (26–30%), *Helianthus annuus* the lowest (19–23%), and *Phragmites australis* falling in between (23–26%). Under water-saving regimes, *Helianthus annuus* fields showed substantially reduced deep percolation (198 mm to 70 mm, 64.6% reduction). *Tamarix chinensis* buffered stress through salt tolerance to sustain transpiration. While *Phragmites australis*’s deep roots secured capillary rise advantage, its 23% *T_a_*/*T_p_* decline highlighted acute sensitivity to water table drawdown. Deep water-saving measures intensify plant water stress and reduce the contribution of groundwater to evapotranspiration, which holds important implications for agricultural water-saving practices, ecological conservation, and water resource management strategies.

## 4. Discussion

### 4.1. Impacts of Intensified Water-Saving Practices on Plant Water Consumption Processes in the Cultivated Land–Wasteland–Lake System

Implementation of intensified water-saving projects in the Hetao Irrigation District has substantially reduced Yellow River diversions. This reduction has led to a continuous decline in the groundwater table. Consequently, the water consumption patterns of the cultivated land–wasteland–lake composite ecosystem have been restructured. This region is characterized by small lakes and mosaic-distributed land covers, with wastelands mainly distributed between cultivated fields and around lake margins, forming a distinctive hydro-ecological unit. Crop evapotranspiration is jointly regulated by meteorological conditions, soil environment, and growth stages, displaying pronounced temporal dynamics. Our SIMDualKc simulations showed that total evapotranspiration of *Tamarix chinensis* during the growing season was 515–525 mm, with capillary rise of 217–252 mm; evapotranspiration of *Helianthus annuus* was approximately 470–473 mm, with capillary rise of 129–191 mm. Compared with Ren et al. [39,40], evapotranspiration and cultivated land capillary rise values were similar, but wasteland capillary rise was lower than their reported 380–394 mm. This difference is attributed to the 4–5 irrigation events in Ren’s study area, which provided lateral subsurface recharge to wastelands, whereas only one irrigation event occurred in our study area, resulting in limited recharge. Against the backdrop of deep water-saving implementation, the decline in groundwater table leads to a reduction in the capillary rise of soil water. This, in turn, causes a decrease in the plant water stress coefficient *(K_s_*) and ultimately results in a drop in *T_a_*/*T_p_*. Although the falling groundwater table reduces soil evaporation (*E*) by lowering surface soil moisture, the magnitude of this reduction exerts a similar impact on both E and evapotranspiration (*ET_c_*_adj_). Consequently, the *E*/*ET_c_*_adj_ ratio remains stable. However, there are fundamental differences in the response strategies among different plant species to cope with this scenario: *Helianthus annuus* relies on artificial irrigation for water redistribution to address water fluctuations; *Tamarix chinensis* maintains its transpiration efficiency through its inherent ability to absorb groundwater and high salt tolerance; *Phragmites australis* primarily depends on water from adjacent lakes to meet its growth requirements. Global studies on evapotranspiration partitioning indicate that soil evaporation typically accounts for 20–40% of total evapotranspiration [37,38], and our *E*/*ET_c_*_adj_ ratios fall within this established range. Notably, wasteland and lake margins also maintained *E*/*ET_c_*_adj_ values within expected levels, confirming their stable role in regional water cycling. Yang et al. [24] reported that groundwater depth in the Shenwu subdistrict increased after intensive water-saving renovations, consistent with our findings. Wang et al. [41] suggested that maintaining groundwater depth within 1.7–2.3 m is optimal for crop growth. Our results show that under current intensive water-saving practices, groundwater fluctuations remain within critical thresholds for plant growth. However, persistent groundwater decline may disrupt this balance, underscoring the need for long-term monitoring of its impacts on plant water use dynamics. Therefore, we recommend integrating agricultural water-saving measures with groundwater regulation, adopting water-adaptive planting and zonal management strategies, and maintaining appropriate groundwater levels in ecologically sensitive areas to support natural vegetation. Moreover, systematic consideration of hydraulic linkages among cultivated land, wasteland, and lakes is essential for developing integrated management strategies that reconcile agricultural production, ecological security, and water resource sustainability, thereby providing a robust scientific basis for achieving harmonious human–water development in the region.

### 4.2. Applicability Analysis of SIMDualKc Model for Multi-Plant Simulation in Cultivated Land–Wasteland–Lake Systems

Existing studies have confirmed the broad applicability of the SIMDualKc model for simulating water dynamics in multi-land-cover systems. For instance, Miao et al. [42] achieved high-accuracy evapotranspiration simulations in spring wheat–*Helianthus annuus* intercropping systems in the Hetao Irrigation District, and their results are consistent with the findings of this study. Li et al. [43] applied the SIMDualKc model in Tongliao, and the results showed that inter-row evaporation of maize under film-free drip irrigation accounted for 26–27% of total evapotranspiration. Zhao et al. [44] quantified the contribution ratio of soil evaporation (17–22%) during the winter wheat growing season in North China. In this study, the soil evaporation fractions (*E*/*ET_c_*_adj_) of the three representative plants remained stable within the range of 19–30%, which aligns well with previous results. Zhao et al. [19] reported that the maximum soil evaporation coefficient (*K_e_*) in winter wheat–summer maize intercropping systems in the North China Plain was 0.8–1.0, whereas our findings show that the maximum *K_e_* value of *Helianthus annuus* was 0.6–0.74, which is lower than this range. This discrepancy is attributed to reduced irrigation under intensified water-saving practices, which limited the effective surface soil moisture supply and further constrained the intensity of soil evaporation. Ren et al. [21] extended the application of the SIMDualKc model to the water consumption of *Tamarix chinensis* in wastelands using the coupled HYDRUS–SIMDualKc model. Our results are consistent with their findings, further validating the model’s applicability in integrated systems comprising cultivated land (*Helianthus annuus*), wasteland (*Tamarix chinensis*), and lakeshore areas (*Phragmites australis*). The findings demonstrate that the SIMDualKc model effectively resolves water transport processes across crops, shrubs, and wetland vegetation, providing a robust tool for investigating water consumption mechanisms in complex ecosystems. However, this study was conducted over three years at a single site with a limited observation range, and the results are also influenced by strong monsoonal seasonal signals. Consequently, it is difficult to fully capture the spatial heterogeneity of hydrological processes at the irrigation–district scale. In addition, due to the current system scale, the quantification of lateral hydrological connectivity between different land parcels remains insufficient. Future studies should integrate remote sensing technology with distributed hydrological models and establish a multi-site monitoring network across the irrigation district. This approach would enable a systematic analysis of the long-term mechanisms of water and salt redistribution in systems with diverse land types under the context of deep water-saving. This study provides essential scientific evidence for advancing water-saving practices in the Yellow River Basin and emphasizes the importance of balancing agricultural and ecological water demands. It further highlights the need to develop adaptive water resource management strategies based on the combination of simulation and monitoring to ensure regional water security and ecological sustainability.

## 5. Conclusions

(1) The model demonstrated strong capability in simulating soil moisture content and estimating crop yield. During the calibration–validation process, the simulated soil moisture values showed good agreement with measured data: Root Mean Square Error (*RMSE*) ≤ 1.0%, Nash–Sutcliffe efficiency (*NSE*) ranged from 0.78 to 0.91, and the coefficient of determination (*R^2^*) ranged from 0.81 to 0.92. These results indicate that the model effectively reproduces soil moisture dynamics and provides reliable estimates of crop yield within the study area.
(2) In 2018 and 2023, the *K_cb_* and *K_cb_*_adj_ curves of *Helianthus annuus*, *Tamarix chinensis*, and *Phragmites australis* largely overlapped, indicating only minor water–salt stress. In contrast, under deep water-saving conditions in 2024, the *K_cb_*_adj_ curves of all three species exhibited a significant decline, reflecting enhanced stress induced by groundwater table decline and surface salt accumulation.
(3) Significant differences were observed in the proportion of soil evaporation to total evapotranspiration (*E*/*ET_c_*_adj_) among different plant types: 19–23% for *Helianthus annuus*, 26–30% for *Tamarix chinensis*, and 23–26% for *Phragmites australis*. A negative correlation was identified between the dominance of plant transpiration and the intensity of soil water loss. Meanwhile, the contribution of capillary rise to total *ET* was relatively high, accounting for 38.61–43.18% in cultivated land, 41.52–48.93% in wasteland, and 38.08–46.57% in lakeshore areas, highlighting the critical role of groundwater recharge in sustaining ecosystem water consumption.
(4) Under deep water-saving conditions, the ratio of actual to potential transpiration (*T_a_*/*T_p_*) declined markedly, by 3–11% for *Helianthus annuus*, 5–12% for *Tamarix chinensis*, and 23% for *Phragmites australis*. At the same time, capillary rise decreased by approximately 10% in cultivated land, wasteland, and lakeshore areas. These results indicate that deep water-saving measures intensified plant water stress and reduced the contribution of groundwater to evapotranspiration, providing important insights for regional water management strategies.

## Figures and Tables

**Table 1:** Soil physical properties of typical sampling points in the study area.

Sampling Point	Soil Layer (cm)	Clay (<0.02 mm)/%	Silt (0.02–0.5 mm)/%	Sand (>0.5–2 mm)/%	Bulk Density/ (g/cm^3^)	Saturated Hydraulic Conductivity (cm/d)	Θ_s_ (Saturated Water Content)
A1	0–300	3.22	45.73	51.04	1.66	19.67	0.31
A2	0–80	2.22	43.08	54.70	1.68	22.10	0.36
	80–300	2.54	7.76	89.70	1.69	230.84	0.33
A3	0–20	5.72	14.47	79.81	1.52	197.76	0.38
	20–300	0.43	5.94	93.63	1.73	315.12	0.31

Note: A1 refers to cultivated land, A2 refers to wasteland, and A3 refers to lake.

**Table 2:** *Helianthus annuus* irrigation system in 2018, 2023, and 2024.

Crop	Year	Irrigation Date (Month/Day)	Irrigation Amount (mm)	TDS (g/L)	Irrigation Method
*Helianthus annuus*	2018	5/23	186	0.60	Border irrigation
		6/19	104	0.57	
		7/2	96	0.61	
	2023	5/27	252	0.61	
	2024	5/23	249	0.59	

**Table 3:** Plant height of crops and natural vegetation at major growth stages in 2018, 2023, and 2024.

Plant	Year	Plant Height (cm)		
		Initial Stage	Middle Stage	Late Stage
*Helianthus annuus*	2018	16	172	154
	2023	17	171	152
	2024	17	169	150
*Tamarix chinensis*	2018	170	170	170
	2023	170	170	170
	2024	170	170	170
*Phragmites australis*	2018	35	151	162
	2023	34	155	160
	2024	37	152	158

**Table 4:** Goodness-of-fit test indicators relative to model calibration and validation.

	Plant	Item	*RMSE* (cm^3^ cm^−3^)	*NSE*	*R^2^*
Calibration (2024)	*Helianthus annuus*	Soil water content	0.01	0.90	0.90
	*Tamarix chinensis*		0.01	0.86	0.86
	*Phragmites australis*		0.01	0.91	0.92
Validation (2023)	*Helianthus annuus*	Soil water content	0.01	0.83	0.83
	*Tamarix chinensis*		0.01	0.78	0.81
	*Phragmites australis*		0.01	0.85	0.87

**Table 5:** Parameters used for calculating the evaporation reduction coefficient *Kr*.

Parameters	Values		
	Site A1	Site A2	Site A3
Field capacity, *θ_fc_* (cm^3^cm^−3^)	0.35	0.38	0.37
Wilting point, *θ_wp_* (cm^3^cm^−3^)	0.11	0.13	0.12
Soil evaporation layer depth, *Z_e_* (cm)	10.00	10.00	10.00
Total evaporable water, TEW (cm)	1.90	2.40	2.40
Readily evaporable water, REW (cm)	0.80	0.20	0.80

**Table 6:** Comparison of simulated and measured crop yields in 2018, 2023, and 2024.

Crop	Year	Yield (t ha^−1^)	
		Simulated	Measured
*Helianthus annuus*	2018	4.4	4.3 ± 0.4
	2023	4.4	4.2 ± 0.5
	2024	4.3	3.9 ± 0.4

## Data Availability

The data are contained within the article.
